# Re-architecture for requirements of academic space development in the universities: design and validitation of RASD questionnaire

**DOI:** 10.1038/s41598-025-85432-1

**Published:** 2025-01-30

**Authors:** Zahra Karimian, Mahmoud Abolghasemi

**Affiliations:** 1 Department of E-Learning in Medical Sciences, Virtual School and Center of Excellence in E-Learning, Shiraz University of Medical Sciences, Shiraz, Iran; 2 Department of Leadership and Educational management, Faculty of Psychology and Educational Sciences, Shahid Beheshti University, Tehran, Iran

**Keywords:** Higher education, University, Requirements, Academic space development, Psychology, Environmental social sciences, Medical research

## Abstract

Universities are the cornerstone of social development and progress; however, it is essential to enhance the academic environment to support this mission. The objective of this research is to identify the *Requirements for Academic Space Development* (RASD) in universities. The present study was conducted in 2022–2023 using a mixed-method with a qualitative-quantitative sequence. The qualitative section was conducted using purposive sampling and semi-structured interviews with 20 experts of Iranian universities. The data were analyzed using content and thematic analysis at three levels of codes (Items), components, and themes (Main concepts), and then, it was examined quantitatively using a survey on 265 faculty members. For data analysis and validate the instrument, the face and content validity (CVI/CVR), reliability and construct validity were examined. Exploratory and confirmatory factor analysis were used to validate the questionnaire using SPSS 24 and Liseral 8.5 software. In qualitative stage, 67 codes, 4 concepts (formal, political, cultural, and scientific), and 14 components were extracted, which resulted in the word “Re-architecture” based on the acronym of the first letter. The content validity of the RASD questionnaire was confirmed with CVI = 0.941 and CVR = 0.940. The overall reliability was 0.962. For construct validity, first, the sample adequacy was confirmed with KMO = 0.928 and the Bartlett test (P < 0.001). In general, the quantitative section also extracted 14 factors that estimated 70.229% of the total variance. The “Happiness” had the highest factor loading (30.35%), followed by “Unity” (6.11%), “Thinking” (4.23%), Refining” (3.87%), “Expediency” (3.22%), and “Interaction” (2.83%) with the highest factor loadings. Universities need a rethinking, a concept that has been addressed in this research as “Re-architecture”. Perhaps the choice of this term is appropriate because we also need changes in universities, by combining science, re-engineering, cultural considerations, and an artistic integration of all factors influencing academic development.

## Introduction

Universities are long-standing academic institutions that have served various functions over time. Initially, they entered the field with an educational mission; however, their focus later shifted towards knowledge production^1^. In subsequent decades, the provision of practical services emerged as a new mission in higher education^2,3^. Nevertheless, the scientific functions of universities did not end there. Many studies have been conducted on the functions and roles of universities in development^4–8^. One of the most challenging critical perspectives on university functions and a comprehensive view of their roles is the five-fold approach proposed by Boyer (1990). In the 1990s, Ernest Boyer called for a rethinking of university functions, emphasizing the unity of all university roles and advocating for a focus on educational functions and innovations in teaching and learning^9^. The increasing specialization of knowledge has led to two major undesirable consequences. First, the development and production of specialized knowledge in universities have resulted in a large volume of scattered information that is difficult to recognize and evaluate. Second, the scientific landscape, which has become strongly oriented towards specialization and fragmentation, has neglected interdisciplinary and inter-professional activities that foster new knowledge^10,11^. Boyer emphasizes that in today’s world, integrating or synthesizing knowledge is crucial. This function helps create a cohesive understanding by linking fragmented pieces of information^9^. Another consequence of the increasing specialization of knowledge in universities is the emergence of a specialized academic language, which has created a significant gap between university discourse and the broader discourse of society. As a result, the scientific community has become distanced from society. It appears that universities and communities are unable to communicate effectively with each other, existing in two different worlds. Boyer refers to this new need as engagement^12^. Although engagement can be applied across all university functions, it holds particular importance for coordinating with the community, fostering mutual understanding with the public, and ensuring that academic discourse does not replace societal discourse^8,12^. Moreover, the impact of higher education on society extends beyond scientific and specialized educational functions or the provision of academic degrees. More significant functions—such as intellectual development, free thinking, and transforming learners into ideal human beings—are central goals of this academic institution^13^. In addition to studies focusing on the diversity of scientific functions within universities^8–12^, some researchers have examined university functions using qualitative approaches. The challenge regarding the quality of scientific activities has become more pronounced in recent decades as both the volume and diversity of faculty members’ activities have increased quantitatively, alongside rising expectations for universities’ roles and responsibilities^14^. Researchers in higher education have sought to define the quality of university scientific functions. According to Diamond and Adams (1991), scholarly activity necessitates a high degree of specialized knowledge, creates a significant impact, leads to advancements in knowledge boundaries, is methodical and repeatable, is documentable, and must be critically evaluated by peers^15^. Schulman (1999) identifies the most important characteristic of evaluating scholarly activities as their capacity for criticism, analysis, evaluation by peers, and universal presentation^16,17^. Glassick and his colleagues (1997) conducted research to define qualitative characteristics of scientific activities. They found that all university functions possess six common characteristics that elucidate their scientific nature: clear goals, adequate preparation, appropriate methods, significant results, effective presentation, and reflective critique^18,19^. In summary, fostering a knowledge-based and scientific environment in universities requires a coordinated set of high-quality functions. The primary questions remain: What are the necessary infrastructures for developing a scientific environment in universities? What indicators can define the criteria for enhancing a university’s scientific environment?.

Essentially, development in organizations, including universities, is a planned effort that is carried out at the top organizational level with the aim of growth and promotion, and extends to lower levels through planned interventions in organizational processes based on behavioral sciences^20^. According to Bolman and Deal (2013), university leaders look at organizations from various mental frameworks. Frameworks, or lenses, are like windows, maps, and tools that show different parts of a complete picture. Essentially, a framework or mental model is a set of ideas and assumptions that a person has in their mind to understand the world around them. Bolman and Deal introduce four mental frameworks or lenses: structural, political, human resources, and symbolic^13^.

*Structural framework* In the structural framework of an organization, the organization is viewed as a “*Machine*,” and it is assumed that the organization is a predictable and controllable environment. Therefore, emphasis is placed on design and planning, role and rule setting, goal and policy setting, prioritization, task division and coordination, financial issues, and budgeting^13,21^.

*Human resources framework* In this approach, the organization is seen as a “*Family*,” and the human resources or employees are important. The knowledge, skills, and attitudes of individuals are important, as well as their emotions, desires, tendencies, and fears. The focus is on what organizations and individuals do for each other. This highlights the relationships between individuals and organizations. Interaction, empowerment, and motivation are important in this category^13,21,22^.

*Symbolic framework* The symbolic framework focuses on issues of meaning and culture. In this approach, the organization is as a *“Sacred temple”*. Rituals and ceremonies, organizational identity, history and background of the organization, stories, culture, and values are at the heart of this approach^23^. Imaging and symbols create a long-term message because through these signs, organization members become unified with each other^13,21,24^.

*Political framework* The political framework sees organizations as a competitive environment, such as *a “Jungle”*. Utility is the main focus in this approach, and competition and struggle for power and superiority are signs of this type of mental framework^13^. Political infrastructure provides the basis for the development of programs by using those who are politically aware. Explanation of programs, understanding the political background, networking, coalition building, negotiation, and balancing the interests of the organization and competitors are some of the things used in this approach^21^. Lorenz et al. (1997) describe the explanatory domains of organizational development and change in 9 dimensions, including ecology, structure, politics, culture, psychology, human resource, functional, Intelligence, and Decision Making^25^. Bouch (2020) believes that educational leaders view universities through six lenses, including bureaucratic or formal, cultural, political, ambiguous, subjective, and collegial. The importance of these lenses is that leaders choose instruments and development methods based on how they see the world^26^.

At a glance, the literature on university functions highlights their evolution from educational institutions to centers of knowledge production and community engagement. Boyer’s five-fold approach emphasizes the need for integration and engagement with society, addressing the growing disconnect between academic discourse and public understanding. Previous studies have attempted to define quality in scholarly activities but often overlook the unique complexities of higher education environments. Frameworks proposed by Bolman and Deal^21–23^ provide valuable insights into organizational dynamics; however, they may not fully capture the distinct challenges faced by universities today. In summary, while existing literature discusses various frameworks for understanding organizational development in universities, there is a significant gap in identifying specific infrastructures and indicators necessary for creating a scientific environment within these institutions. Current models often overlook the unique complexities and multidimensional nature of universities, which require tailored approaches to foster academic space development.

Addressing this gap is critical as universities face increasing pressures to adapt to rapidly changing societal needs and expectations. Understanding the specific components required for developing a scientific environment can guide university leaders in implementing effective strategies that enhance knowledge production and community engagement. By utilizing Bolman and Deal’s^23^ frameworks alongside Lorenz et al.‘s^25^ dimensions of organizational development, this study aims to provide a comprehensive understanding of how universities can navigate their unique challenges.

The significance of this research lies in its potential to inform educational leaders about the necessary infrastructures for fostering a dynamic academic environment. By identifying and evaluating the components essential for academic space development, this study contributes to the broader discourse on higher education reform. Furthermore, it emphasizes the importance of aligning university functions with societal needs, ultimately promoting intellectual development, free thinking, and community engagement. The findings will not only enhance academic discourse but also provide practical insights for university administrators seeking to implement effective organizational strategies in today’s complex educational landscape.

Although the patterns presented in organizational development models are applicable to all organizations today, universities—due to their complexity, multidimensionality, and exposure to a diverse group of high-level human resources—require the discovery of a pattern that is appropriate to their unique characteristics; a pattern that can align with the turbulent conditions of today’s world. In this study, answers were provided to three major questions:

- Identifying the components for Requirements of Academic Space Development in universities.
- Psychometric evaluation of Requirements of Academic Space Development questionnaire.
- Determining the importance Requirements of Academic Space Development components.

## Methods

### Research design

The research conducted with mixed-methods in 2022–2023. The main design of this study was sequential exploratory and of the instrument development model. In the sequential exploratory design, the researcher first tries to gain a deep understanding of the phenomenon and then seeks to generalize the understanding to larger samples^27^.

Since there were few similar studies and no theoretical or instrument foundations for measuring variables related to scientific development infrastructure, it was necessary to first identify the components of scientific development infrastructure using a qualitative approach, and then test hypotheses derived from the qualitative data using a quantitative approach with the developed instrument.

In this study, the qualitative phase aimed at identifying the items, components, and themes of the instrument was conducted through semi-structured interviews with experts from diverse fields of medical sciences, humanities, basic sciences, and engineering. After data collection, thematic content analysis was performed, leading to the extraction of items, components, and themes, and the development of a model instrument. In the quantitative phase, model validation was carried out based on a survey administered to faculty members in both medical and non-medical disciplines. Initially, a psychometric approach was used to validate the tool, and the significance of the components was also examined.

### Population and samples

The statistical population for the qualitative section consisted of experts in higher education. A purposive sampling method was employed to select these individuals. In selecting the samples, efforts were made to include individuals with significant experience across all scientific activities, including education, research, executive and service roles, and community engagement. The selected experts not only possessed a strong scientific reputation but also demonstrated effective managerial skills. To ensure maximum diversity among the samples, diversity in both university affiliation and academic field was a key criterion for selection. Initially, a list of experts was compiled from four areas of higher education: technical and engineering, humanities, basic sciences, and medical sciences. Interviews with the selected participants continued until data saturation was achieved. In total, 20 informed experts participated in the interviews concerning issues related to higher education.

In the quantitative section of the study, the statistical population consisted of approximately 1400 faculty members at two university: Shiraz University of Medical Sciences (SUMS) and Shiraz University, who were randomly selected in proportion to each faculty. The inclusion criteria for all faculty members were having at least three years of work experience and consenting to participate in the study. The exclusion criterion was questionnaires with more than 20% unanswered questions. The sample size was estimated to be around 302 participants, based on the Cochran formula^28^ with a 95% confidence interval and a statistical error estimate of 0.05. In this formula, P and Q values were considered as 0.5, Z value was 1.96, and N was the total number of the statistical population.

$$n = \frac{{\frac{{z^{2} pq}}{{d^{2} }}}}{{1 + \frac{1}{N}\left[ {\frac{{z^{2} pq}}{{d^{2} }} - 1} \right]}}$$

### Data collection and instrument

*Qualitative instrument* To identify the components for *Requirements* of *Academic Space Development* (RASD). the study commenced with a review of the theoretical foundations and research, and then data was collected through semi-structured in-depth interviews with participants. The questions were raised in face-to-face meetings with participants at a pre-arranged time. Prior to the interviews, the time of the interviews was arranged with participants via in-person contact or email, taking into account their available time. The list of questions was sent to the participants via email so that they could review the overall topic of the study if they wished to. One main question and two probing questions were asked, and additional following questions were asked based on the responses.

- What are the necessary infrastructures for the development of the scientific space of the university in your opinion?
- What features are essential for creating a scientific space in universities?
- What are the damages of the current scientific space of the university and what are the solutions to overcome these problems?”

The interviews were recorded using a voice recorder, and notes were taken simultaneously. In instances where participants were unwilling to be recorded, all conversations were documented in detail. The duration of each interview ranged from 60 to 120 min, with an average length of 100 min. The start and end times of the interviews were determined by the participants. If an interviewee preferred to postpone part of the discussion to a later time, it was rescheduled and completed in a subsequent meeting.

After each interview, data implementation began within less than 12–30 h, and with repeated review of the participants’ statements, the essence of their speeches was recorded, taking into account pauses, emotions, emphasis, and the participants’ facial expressions. It should be noted that during data collection and recording, the expressed content of the participants was fully and descriptively presented. Burns and Grove (1999) believe that these narratives should not be fragmented and coded during the interview section, as this would break down the data and destroy its meaning^29^. Therefore, in the implementation and writing of the interview content, each question was first asked, and then the narrative of each participant regarding that question was written. Then, in each interview, the main and evident points that had an impact on coding were underlined. The researcher inferred the explicit and implicit content of words in the form of phrases.

*Validity and reliability of the qualitative section instrument* To ensure the validity of the work in the qualitative section, Lincoln and Guba’s criteria including credibility, dependability, confirmability, and transferability were used^30^. The triangulation was used in this research and a combination of qualitative and quantitative data was examined and analyzed both quantitatively and qualitatively. In selecting the samples, diversity in the fields of the participants was considered. In selecting the qualitative research samples, a diverse range of criteria, such as reputation, scientific and managerial background, relevant scientific and executive experiences, were taken into account. Coding was done by two researchers. After converting the interviews and audio files into text and finalizing the interviews, a version of the file was sent to the interviewees for final confirmation of their interpretation. After that, open codes, components and main concept were categorized by content analyzing. The content analysis results were reviewed by three peers with medical education and higher education management backgrounds. In addition, the construct validity was established using factor analysis in quantitative section.

*Quantitative instrument* After extracting concepts and preparing the items, a questionnaire with 67 questions in a six-point Likert scale was prepared. The value of the options was set from completely agree = 6 to completely disagree = 1. The formal and content validity of the questionnaire was checked with the opinions of 10 educational experts and psychometricians. In face validity, 12 sentences needed grammatical editing and simplification, which were corrected and confirmed. Then, content validity was assessed using the Content Validity Index (CVI) indicator. According to Waltz & Bausell’s formula, experts evaluate each question of the questionnaire in terms of relevance, simplicity, and clarity on a scale of 1 to 4, and only options 3 and 4 are entered into the formula to calculate the content validity score. In the CVI method, the acceptable value depends on the number of participants who have chosen options 3 and 4, and the level of agreement between them. In this formula, a minimum of 0.79 agreement among opinions is expected^31^.

$${\text{CVI}} = \frac{{{\text{Number of raters giving a rating of 3 or 4}}}}{{{\text{Total number of raters}}}}$$

Additionally, Content Validity Ratio (CVR) was measured. In this method, the necessity of each question was measured on a three-level spectrum of “essential,” “useful but not essential,” and “not essential.” The following formula was used in this method, where N is the total number of participants and n is the number of individuals who have selected the “essential” option^32^.

$$CVR = \frac{{n - \left( {\frac{N}{2}} \right)}}{{\frac{N}{2}}}$$

*Reliability* To determine the reliability of the questionnaire, the internal consistency of the questions was used, and the Cronbach’s alpha was calculated. In this formula, the total reliability coefficient of the test is equal to r_a_, the variance of the scores of each section of the test is equal to σ^2^ j, the number of sections of the test is equal to j, and the variance of the total scores of the test is equal to σ2.

$$r_{a} = \frac{j}{{j - 1}}\left( {1 - \frac{{\sum {\sigma _{j}^{2} } }}{{\sigma ^{2} }}} \right.$$

After final adjustments, the questionnaire was sent to the participants via email, and a reminder email was sent approximately three times.

### Data analysis

*Qualitative data analysis* Qualitative data analysis was conducted using semi-structured and in-depth interviews, and a combination of qualitative and quantitative content analysis methods were used to extract concepts from the interviews. The data were categorized into three levels of: coding (Items), components, and themes main concepts. During the content analysis process, the researcher frequently noted MEMOs or mental reflections, and concepts and components were extracted through an iterative process. In response to the question related to identifying the infrastructures for the development of the scientific environment, after reducing and eliminating data, approximately 165 open codes were initially extracted, which were summarized and reduced to a total of 67 final items. Based on the apparent and meaningful content similarities, theoretical foundations, and a logical reasoning system, the codes were categorized into four main categories, 14 core components, and 67 items.

*Quantitative data analysis* Quantitative data analysis was conducted using the SPSS 24 software. After conducting exploratory factor analysis, we arrived at four main categories that were consistent with the general categories of the qualitative section (formal, cultural, scientific, and political) and with the organizational development theory proposed by Bolman and Deal (2013) (structural, symbolic, human resources, and political)^13^. Therefore, in the next step, confirmatory factor analysis was performed.

*Ethics* All procedures were performed in accordance with the applicable guidelines and regulations of the Research Deputy of SUMS, with the approved project number 27,973 and confirmed by the Research Ethics Committee with code IR.SUMS.REC.1402.063.

## Results

### Demographic characteristics

In Table 1 the demographic characteristics of research samples in qualitative and quantitative sections have been shown (Table 1).

**Table 1:** Demographic characteristics of participants in qualitative and quantitative sections.

Characteristics of higher education experts in qualitative section								
Code	Field of study	Years of experience	Age range	Academic rank				
				Professor	Associate Prof.	Assistant Prof.		
01–05	Technical and Engineering Sciences	17–40	50–70	4	1	0		
06–10	Basic Sciences	22–35	52–62	4	1	0		
11–15	Humanities	21–36	49–71	3	2	0		
16–20	Medical Sciences	13–18	43–51	3	1	1		

Characteristics of faculty members participated in quantitative section								
Characteristics		None Medical University (Shiraz University)			Medical University (SUMS)			
		Engineering	Basic sciences	Humanities	Clinical medicine	Medical basic sciences	Para-medical sciences	Total
Gender	Female	2 (1%)	9 (% 3)	9 (% 3)	13 (% 5)	15 (% 6)	26 (% 10)	74 (28%)
	Male	47 (%18)	37 (% 14)	38 (14%)	31 (12%)	25 (9%)	13 (% 5)	191 (72)
	Total	49 (% 19)	68 (% 17)	47 (% 18)	44 (% 17)	40 (% 15)	39 (% 15)	265 (%100)
Academic rank	Instructor	0 (0%)	3 (1%)	2 (1%)	0 (0%)	2 (1%)	26 (10%)	33 (12%)
	Assistant Professor	19 (7%)	32 (12%)	15 (6%)	24 (9%)	14 (5%)	10 (4%)	114 (43%)
	Associate Professor	17 (6%)	7 (3%)	16 (6%)	12 (5%)	16 (6%)	3 (1%)	71 (27%)
	Professor	13 (5%)	4 (2%)	14 (5%)	8 (3%)	8 (3%)	0 (0%)	47 (18%)
	Total	49 (% 18)	46 (% 17)	47 (% 18)	44 (% 17)	40 (% 15)	39 (% 15)	265 (%100)
Teaching experience	3–10 years	21 (8%)	19 (7%)	14 (5%)	22 (8%)	11 (4%)	16 (6%)	103 (39%)
	11–20 years	13 (5%)	17 (6%)	13 (5%)	14 (5%)	20 (8%)	18 (7%)	95 (36%)
	> 21 years	15 (6%)	10 (4%)	20 (8%)	8 (3%)	9 (3%)	5 (2%)	67 (25%)
	Total	49 (18%)	46 (17%)	47 (18%)	44 (17%)	40 (15%)	39 (15%)	265 (%100)

The qualitative section of the research indicates that the samples were drawn from four fields: medical sciences, humanities, basic sciences, and engineering. Five participants from each field were included in the study. Additionally, there was a significant emphasis on diversity regarding age, gender, academic rank, and work experience among the participants. In the quantitative section, findings revealed that the majority of individuals held the rank of associate professor (43%), and most participants had work experience ranging from 11 to 20 years (36%).

### Qualitative finding

#### Identification of RASD components

To identify the components and themes of RASD, content and thematic analysis was performed based on the experts’ viewpoints. The components were extracted based on exploratory content analysis of the expert opinions provided in response to all questions at the sentence, phrase, paragraph, and thematic levels. Based on the results, the infrastructures for RASD were initially extracted from 165 open codes or primary indicators and were then reduced and consolidated to 67 open codes (Items). The content analysis of the experts’ opinions was performed through a continuous iterative process, and memoing played a significant role in this regard. Memoing is a type of mental reflection by the researcher that is not based on any specific rule and can occur differently in each researcher’s mind, indicating the researcher’s mental process in analyzing and categorizing the data^33^.

The comparative analysis and the interpretation of the experts’ viewpoints showed that the participants considered a set of tangible and hard factors (resources, facilities, evaluation and promotion mechanisms, regulation and law revision, quality improvement, etc.) and intangible soft factors (knowledge, attitude, culture, value creation, planning and policy, happiness, critique, interaction, etc.) necessary for the development of a scientific environment. Therefore, the infrastructures of a scientific environment comprise a combination of cultural, scientific, political, and structural measures. To develop and change the university, attention must be paid not only to the structure, and form but also to the value, cultural, and knowledge dimensions. The combination of these concepts evokes a term that can encompass both structural engineering and values and culture, which researchers referred to as “Architecture” during the sub-categorization process (Table 2).

**Table 2:** Components arrangement of RASD based on content analysis of experts’ view.

*R*	E	A	*R*	C	H	I	T	E	C	T	U	*R*	E
Resources	Evaluation	Autonomy	Refining	Cultural symbols	Happiness	Interaction	Thinking	Education	Critique	Tactics	Unity	Regulation	Expediency

Based on Table 2, after extracting the categories, we identified 14 components. To align the extracted components, we matched the intermediate coding or components with the acronym ‘Rearchitecture’.

Finally, the extracted pattern with **67** open codes (Items) resulted in 14 components and 4 main categories (Table 3).

**Table 3:** Requirements of academic space development based on content analysis of experts’ viewpoint.

Components	Codes (Items)	F
Concept1: Formal		
Refining	Systematic connection of professors’ activities with quality optimization programs	12
	Harmonization of scientific functions in the university towards quality improvement	16
	Review and refinement of current laws, regulations, process and structures	10
	Existence of new necessary policies for academic development and promotion	14
	Critique and review of academic functions through interaction with social institutions	9
Resource	Allocation of sufficient resources and budget for the academic development and promotion of professors	8
	Strengthening facilities and resources in university for academic development (Laboratories, Libraries, e-Infrastructures)	12
	Development of new e-Infrastructures and technologies for the development and dissemination of knowledge	16
Regulation	Existence of clear regulations and guidelines for the development of academic space	12
	Valuation and accreditation of participation in academic promotion programs	12
	Existence of counseling and support centers for quality development in the university	13
	Existence of mechanisms for preserving and documenting managerial and educational experiences	11
	Existence of mechanisms for documenting and preserving the experiences of pioneers	9
	Existence of laws and mechanisms for preserving individuals’ intellectual property	7
	Existence of structures such as think tanks and brainstorming chairs	8
Evaluation	Existence of mechanisms for evaluating and encouraging educational and academic innovations	17
	Existence of motivating regulations and mechanisms for evaluation of academic development	16
	Orientation of evaluation mechanisms towards accountability and academic authenticity	11
	Comprehensive attention to all academic functions (Avoidance of one-dimensional emphasis on research)	14
	Existence of a comprehensive and multidimensional scientific evaluation system	14
Concept2: Political		
Expediency	Negotiation and compromise skills of administrator in decision-making meetings	5
	Entrepreneurial skills and attraction of financial resources outside the university	10
	Ability to establish win-win interactions with external organizations	9
	Positive use of influential individuals’ credibility and experience in facilitating activities	11
Tactics	Presence of strategic and innovative managers in the organization to take advantage of opportunities	4
	Effective communication of managers with their power sources to facilitate administrative processes	4
	Ability to present and reflect the organization’s capabilities by managers	3
	Ability to attract agreement and convergence of opinions in discussions and negotiations	5
Autonomy	Power of specialized fields in university management and decision-making	12
	Scientific and managerial independence in attracting and directing resources	20
	Existence of open space for criticism and exchange of opinions and ideas	20
	Power of educational groups in scientific and managerial decision-making	9
	Presence of faculty members in the group or faculty among senior university managers	3
Concept3: Scientific		
Interaction	Members of the academic faculty’s tendency to participate in team activities	18
	Management decision-making based on interaction and participation	17
	Willingness and positive feeling to share knowledge and experiences with others	14
	Appropriate interaction between senior and newly hired academic faculty members	14
	Interdisciplinary communication between educational groups	16
	Exchange of knowledge and feedback between the university and industry	18
	International relations and links with scientific networks beyond borders	17
Education	Holding teacher training program and promotion courses for academic members	17
	Existence of scientific meetings for sharing academic and educational experiences	15
	Active participation in national and international scientific conferences	13
	Active participation of faculty members in scientific courses abut new concept and technologies	18
	Access to resources, books, scientific databases, etc. for knowledge acquisition	14
Thinking	Scientific thinking and professional ethics governing university functions	18
	Prioritizing correct thinking over opportunism and conservatism	15
	Embracing new ideas and thoughts and avoiding stagnation and routine	14
Critique	Presence of a critical spirit and proper critique in the scientific community	20
	Availability of appropriate space for criticism to challenge scientific knowledge and experiences	20
	Feeling of academic freedom of expression in exchanging opinions and criticizing ideas	20
	Attention to the necessity of critique in all scientific university functions	19
	Avoidance of scientific isolation and indifference to community issues	12
Concept4: Cultural		
Cultural symbols	Tendency to share knowledge and experiences through media and social networks	5
	Existence of ceremonies and rituals for appreciating innovations and best practices	15
	Holding festivals or special awards at the university and national levels	8
	Individuals’ tendency to engage in joint and collective academic activities	10
	Trust and solidarity among individuals in exchanging knowledge and experiences	17
	Encouraging the culture of sharing and distributing information with others in the organization	18
Unity	Feeling of belonging and proud in the university as part of personal identity	11
	Feeling of belonging and solidarity with the university’s achievements and successes	8
	Orientation of academic activities of all university professors to achieve university excellence	16
	Prioritizing university interests and community excellence over personal achievements	17
Happiness	Presence of scientific enthusiasm and strengthening positive spirit in the university	8
	Strong hope and motivation for scientific development and progress among professors	9
	The impact of celebrations, rituals, and ceremonies for appreciating professors on scientific enthusiasm	8
	The presence of leading and motivated persons to create spirit of hope and enthusiasm in academic environment	10

Based on Table 3 that showed the thematic content analysis of interviews, four overarching concepts—formal, cultural, scientific, and political infrastructures—were extracted, comprising 14 components and 67 items. The frequency of the items is also presented in this table.

**Fig. 1:**
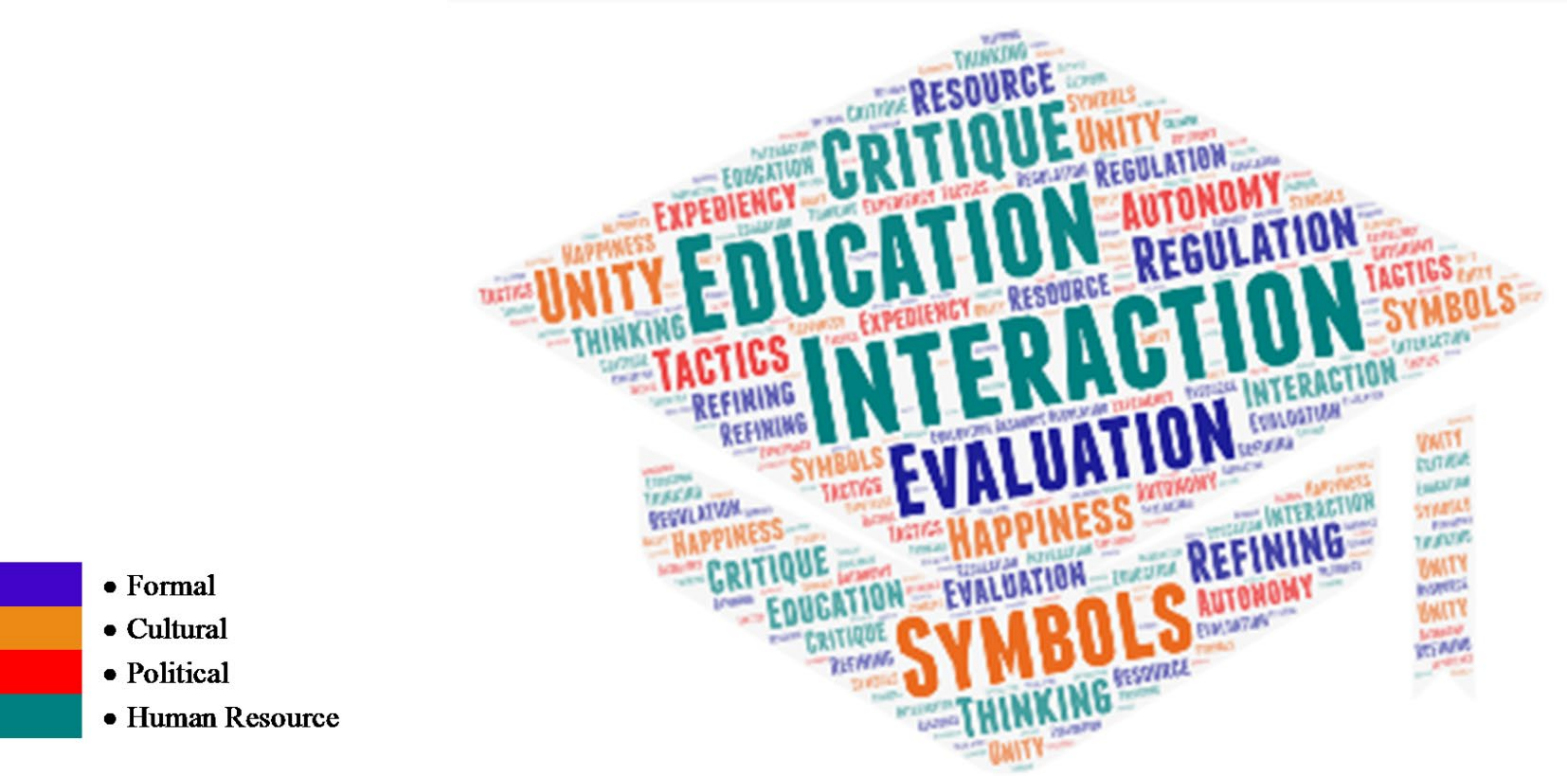
Framework and weight of extracted components based on content analysis of experts’ views.

Also, the summary of Table 3 is presented in Fig. 1. In this Fig. 1, the number of times a component is repeated has been entered as the weight of opinions on the clued based website. As the results show, the higher education experts have emphasized on components such as interaction, development of critical space, education and empowerment, proper and objective evaluation, cultural symbols, unity, etc. in this visual representation. Overall, this diagram indicates that the focus of higher education experts is on the development of the scientific environment, improvement of the quality of education and research, proper and objective evaluation, and strengthening of culture and unity (Fig. 1).

### Quantitative findings

#### Validation of the RASD

*Content validity* Since the components and items of the questionnaire to measure the RASD were developed for the first time, it was necessary to validate the questionnaire. The results of content validity showed that both the Content Validity Index (CVI) and Content Validity Ratio (CVR) were at an appropriate level (CVI = 0.941, CVR = 0.940) (Table 4). As can be seen in the columns related to CVI, some items had a value of less than 0.7, but their CVR score was appropriate, so we did not remove them until the final decision was made on the construct validity. However, we revised the items in terms of wording (simplification and clarification) and they were retained after approval by experts in the questionnaire.

*Reliability* Regarding the internal consistency of the questions, the Cronbach’s alpha value of 0.962 is excellent, and the internal consistency of the items have been confirmed. Furthermore, the Cronbach’s alpha of Sub-components indicates the suitability of the questionnaire. The details of the content validity and reliability (internal consistency of the questions) of the questionnaire are presented in Table 4.

**Table 4:** Psychometric properties of content validity and reliability of the RASD questionnaire N include = 210.

Concepts	Components	Items		Content validity				Reliability		Concepts	Components	Items	Content validity				Reliability	
				CVR	CVI			Cronbach’s Alpha					CVR	CVI			Cronbach’s Alpha	
				Essential	Simple	Clear	Relevant	Factors	If item deleted				Essential	Simple	Clear	Relevant	Factors	If item deleted
Formal	Refining	Q01		0.8	0.9	1.0	1.0	0.879	0.961	Scientific	Interaction	Q34	1.0	1.0	1.0	1.0	0.826	0.961
		Q02		0.8	0.9	0.9	1.0		0.961			Q35	1.0	1.0	1.0	1.0		0.961
		Q03		1.0	0.8	0.6	1.0		0.961			Q36	1.0	1.0	1.0	0.9		0.962
		Q04		1.0	0.9	0.8	1.0		0.961			Q37	1.0	1.0	1.0	1.0		0.961
		Q05		1.0	0.7	0.6	1.0		0.961			Q38	1.0	1.0	1.0	1.0		0.961
	Resource	Q06		1.0	1.0	0.9	1.0	0.602	0.961			Q39	1.0	1.0	1.0	1.0		0.962
		Q07		1.0	1.0	1.0	1.0		0.961			Q40	1.0	1.0	1.0	1.0		0.960
		Q08		1.0	1.0	1.0	1.0		0.961		Education	Q41	1.0	1.0	1.0	1.0	0.737	0.961
	Regulation	Q09		1.0	1.0	1.0	1.0	0.787	0.961			Q42	1.0	1.0	0.9	1.0		0.961
		Q10		0.8	1.0	1.0	1.0		0.961			Q43	1.0	1.0	1.0	0.9		0.962
		Q11		0.8	1.0	1.0	1.0		0.961			Q44	1.0	1.0	1.0	1.0		0.961
		Q12		1.0	0.8	0.9	1.0		0.961			Q45	1.0	1.0	1.0	1.0		0.961
		Q13		1.0	1.0	1.0	0.8		0.961		Thinking	Q46	1.0	1.0	1.0	1.0	0.600	0.961
		Q14		1.0	0.8	1.0	0.8		0.961			Q47	1.0	1.0	0.9	1.0		0.961
		Q15		1.0	0.8	0.7	0.9		0.961			Q48	1.0	1.0	1.0	1.0		0.961
	Evaluation	Q16		1.0	0.7	1.0	1.0	0.705	0.961		Critique	Q49	1.0	1.0	1.0	1.0	0.753	0.961
		Q17		1.0	0.8	1.0	1.0		0.961			Q50	1.0	0.8	0.9	1.0		0.960
		Q18		1.0	0.8	0.8	0.8		0.961			Q51	1.0	1.0	1.0	1.0		0.961
		Q19		1.0	0.7	0.7	1.0		0.961			Q52	1.0	0.9	0.9	1.0		0.962
		Q20		1.0	0.7	0.7	1.0		0.961			Q53	1.0	0.9	0.8	1.0		0.961
Political	Tactics	Q21		0.6	0.8	0.7	1.0	0.786	0.961	Cultural	Cultural symbols	Q54	1.0	1.0	1.0	0.9	0.636	0.961
		Q22		0.8	0.9	0.9	1.0		0.961			Q55	1.0	1.0	1.0	1.0		0.961
		Q23		0.6	0.8	0.8	0.7		0.961			Q56	1.0	1.0	1.0	1.0		0.961
		Q24		0.6	0.9	0.9	0.8		0.961			Q57	1.0	1.0	1.0	1.0		0.962
	Expediency	Q25		1.0	0.8	0.8	1.0	0.864	0.961			Q58	1.0	1.0	1.0	0.8		0.961
		Q26		0.6	0.6	0.7	0.8		0.961			Q59	1.0	1.0	1.0	1.0		0.962
		Q27		1.0	1.0	1.0	1.0		0.961		Unity	Q60	1.0	1.0	1.0	0.7	0.834	0.961
		Q28		0.6	1.0	1.0	0.7		0.961			Q61	0.6	1.0	1.0	0.7		0.961
		Q29		1.0	1.0	1.0	1.0		0.961			Q62	1.0	1.0	1.0	1.0		0.961
		Q30		1.0	1.0	1.0	1.0		0.961			Q63	0.8	1.0	1.0	1.0		0.961
	Autonomy	Q31		1.0	1.0	1.0	1.0	0.725	0.961		Happiness	Q64	1.0	1.0	1.0	1.0	0.823	0.960
		Q32		1.0	1.0	1.0	1.0		0.961			Q65	1.0	0.9	0.9	1.0		0.960
		Q33		1.0	0.9	0.9	1.0		0.962			Q66	1.0	1.0	1.0	1.0		0.961
												Q67	0.6	1.0	0.8	1.0		0.961
CVI _Relevance_ =0.958			CVI _Simplicity_ =0.933			CVI _Clarity_ =0.931		CVI _Total_ =0.941			CVR _Total_ = 0.940		Reliability _Total_ = 0.962					

Based on Table 4, the overall CVI is 0.931, the CVR is 0.940, and the internal consistency of the questions, measured by Cronbach’s alpha, is 0.962. These values indicate that the instrument demonstrates very good validity and reliability.

*Construct validity* Both exploratory factor analysis (EFA) and confirmatory factor analysis (CFA) were used to determine the construct validity and factor loading of each component. As the components had a strong resemblance to the nature of the items, according to the theory of Bolman and Deal^13^, CFA was used for re-examined and confirm the factor analysis results.

### Exploratory factor analysis

*Criteria for conducting EFA* In order to determine the appropriateness of using EFA for this research and sample adequacy, the KMO value and the significance of the Bartlett’s test of sphericity were examined^34^. Based on the result presented in Table 5, the sample size was sufficient to use an appropriate factor analysis method (KMO = 0.897) and (*p* < 0.001), indicating a very high goodness the factor analysis in this study (Table 5).

**Table 5:** KMO and Bartlett’s test for determining RASD questionnaire.

Kaiser-Meyer-Olkin measure of sampling adequacy	Bartlett’s test of sphericity		
	Sig	df	χ^2^
KMO = 0.897	0.0001	2211	9027

Definition of KMO score ranges in exploratory factor analysis			
0 < KMO < 0.49 Unacceptable	0.50 < KMO < 0.59 Weak	0.60 < KMO < 0.69 Medium	
0.70 < KMO < 0.79 Acceptable	0.80 ≤ KMO ≤ 0.89 Appropriate	0.90 < KMO < 1.00 Excellent	

*Criteria for variable suitability after conducting factor analysis* After confirming the goodness of fit for the factor analysis, and to determine the appropriate variables to remain in the research, the factor loading of each variable was determined. In this study, variables with a factor loading greater than 0.5 were considered suitable for remaining in the research (Table 6).

**Table 6:** Communalities matrix of each variable of RASD questionnaire.

No.	Extraction	No.	Extraction	No.	Extraction	No.	Extraction
Q01	0.733	Q18	0.753	Q35	0.657	Q52	0.653
Q02	0.734	Q19	0.709	Q36	0.609	Q53	0.656
Q03	0.791	Q20	0.738	Q37	0.770	Q54	0.589
Q04	0.708	Q21	0.747	Q38	0.713	Q55	0.737
Q05	0.712	Q22	0.655	Q39	0.767	Q56	0.691
Q06	0.647	Q23	0.737	Q40	0.710	Q57	0.660
Q07	0.681	Q24	0.709	Q41	0.737	Q58	0.705
Q08	0.725	Q25	0.704	Q42	0.721	Q59	0.801
Q09	0.702	Q26	0.736	Q43	0.591	Q60	0.596
Q10	0.716	Q27	0.770	Q44	0.767	Q61	0.747
Q11	0.768	Q28	0.767	Q45	0.646	Q62	0.775
Q12	0.731	Q29	0.646	Q46	0.647	Q63	0.765
Q13	0.686	Q30	0.647	Q47	0.649	Q64	0.712
Q14	0.773	Q31	0.622	Q48	0.565	Q65	0.727
Q15	0.640	Q32	0.728	Q49	0.671	Q66	0.686
Q16	0.683	Q33	0.605	Q50	0.783	Q67	0.701
Q17	0.763	Q34	0.622	Q51	0.752	*	*

Based on the results presented in the Table 6, all 67 items related to the RASD questionnaire have a factor loading greater than 0.5, indicating that all variables are suitable for remaining in the research, as determined by exploratory factor analysis.

**Fig. 2:**
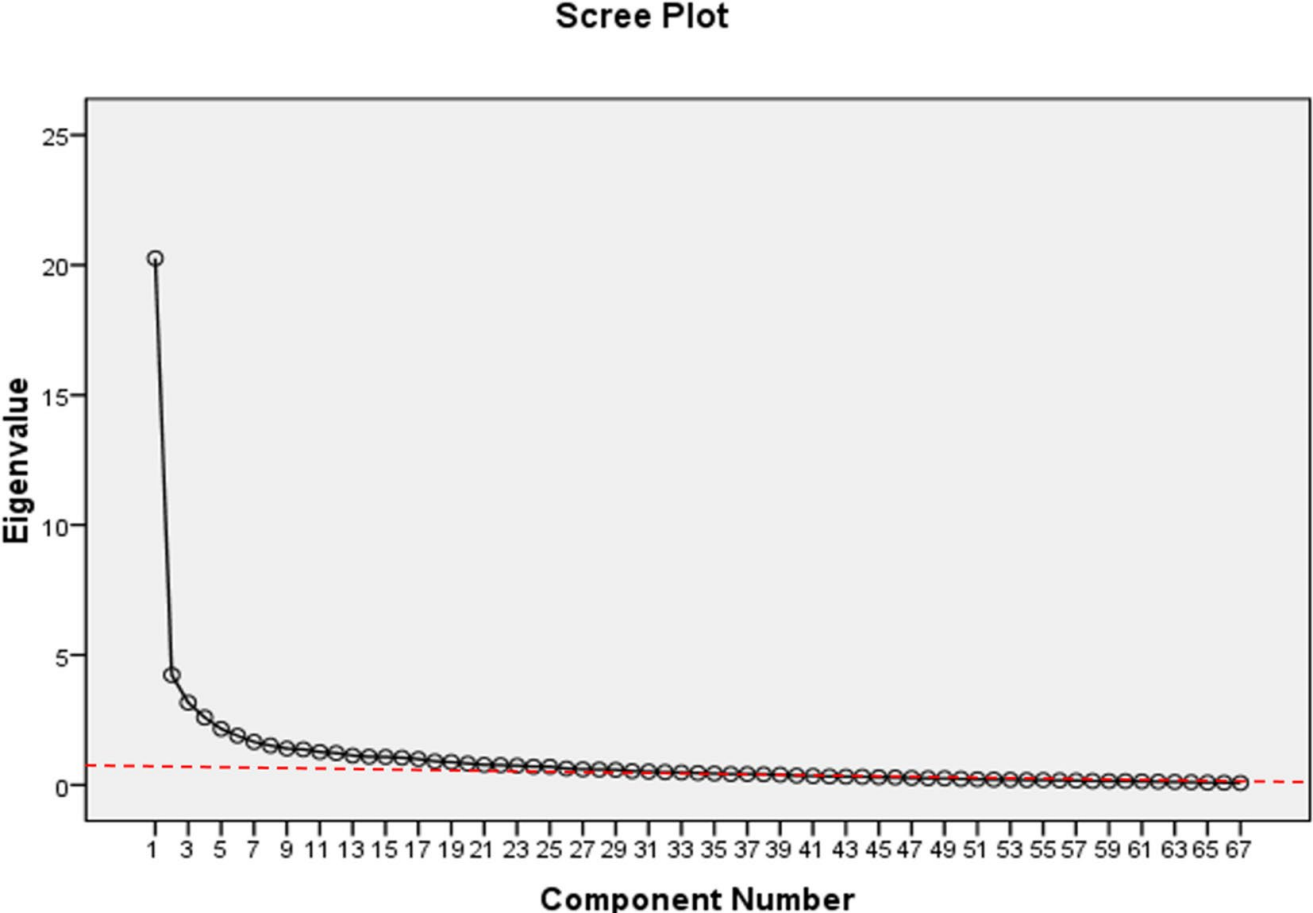
Scree plot resulting from the exploratory factor analysis of RASD questionnaire.

*Determining the number of factors* In order to determine the number of variables related to the RASD and identify the principal components, Kaiser’s criterion was used. According to the Kaiser criterion, only factors with an eigenvalue greater than 1 are acceptable. Based on this criterion, 16 factors were determined by exploratory factor analysis. According to the results presented in the Table 7, the 16 extracted factors explain a total of 70.23% of the variance of RASD. (Table 7; Fig. 2).

**Table 7:** Extracted factors from EFA and the amount of variance explained by each factor.

Factors	Initial eigen values			Extraction sums of squared loadings			Rotation sums of squared loadings		
	Total	% of variance	Cumulative %	Total	% of variance	Cumulative %	Total	% of variance	Cumulative %
1	20.257	30.235	30.235	20.257	30.235	30.235	6.127	9.144	9.144
2	4.228	6.311	36.546	4.228	6.311	36.546	5.818	8.683	17.827
3	3.165	4.723	41.269	3.165	4.723	41.269	4.190	6.254	24.081
4	2.595	3.873	45.142	2.595	3.873	45.142	3.502	5.227	29.308
5	2.158	3.220	48.362	2.158	3.220	48.362	3.426	5.114	34.422
6	1.893	2.826	51.188	1.893	2.826	51.188	3.201	4.778	39.200
7	1.655	2.471	53.659	1.655	2.471	53.659	3.101	4.629	43.829
8	1.514	2.259	55.918	1.514	2.259	55.918	3.077	4.592	48.420
9	1.396	2.084	58.002	1.396	2.084	58.002	2.692	4.018	52.439
10	1.368	2.041	60.043	1.368	2.041	60.043	2.045	3.052	55.490
11	1.268	1.893	61.936	1.268	1.893	61.936	2.043	3.049	58.540
12	1.225	1.829	63.765	1.225	1.829	63.765	1.878	2.803	61.343
13	1.128	1.683	65.449	1.128	1.683	65.449	1.706	2.546	63.889
14	1.085	1.619	67.068	1.085	1.619	67.068	1.568	2.340	66.229
15	1.072	1.600	68.667	1.072	1.600	68.667	1.378	2.057	68.286
16	1.046	1.562	**70.229**	1.046	1.562	70.229	1.302	1.943	70.229
17	0.994	1.483	71.712						

* Cummulitative percentage of variance explained in eigen value point.

In the qualitative section and based on the theoretical concepts, logical arguments and content analysis of interviews with higher education experts, 14 factors were extracted. Two questions with only one item were combined with other related factors based on the researcher’s inference from the item and theoretical foundations, resulting in a final questionnaire with 14 items. Among the 14 factors, the “Happiness” factor had the highest factor loading (30.35%), followed by “Unity” with a factor loading of (6.11%), “Thinking” (4.23%), Refining” (3.87%), “Expediency” (3.22%), and “Interaction” (2.83%) with the highest factor loadings. Total variance explained of RASD questionnaire items is shown in Table 8.

**Table 8:** Total variance explained of RASD questionnaire items.

Rank	Items	r	Rank	Items	r
1. Happiness	Q64	0.612			
	Q65	0.646	8. Cultural symbols	Q54	0.466
	Q66	0.465		Q55	0.688
	Q67	0.607		Q56	0.619
2. Unity	Q60	0.635		Q57	0.717
	Q61	0.743		Q58	0.592
	Q62	0.777		Q59	0.786
	Q63	0.724	9. Critiques	Q49	0.414
3. Thinking	Q46	0.424		Q50	0.406
	Q47	0.394		Q51	0.350
	Q48	0.567		Q52	0.679
4. Refining	Q01	0.689	10. Tactics	Q21	0.655
	Q02	0.760		Q22	0.547
	Q03	0.798		Q23	0.621
	Q04	0.724		Q24	0.785
	Q05	0.639		Q25	0.631
5. Expediency	Q25	0.431	11. Resources	Q06	0.340
	Q26	0.726		Q07	0.586
	Q27	0.666		Q08	0.434
	Q28	0.704	12. Education	Q41	0.739
6. Interaction	Q34	0.497		Q42	0.526
	Q35	0.506		Q43	0.696
	Q36	0.668		Q44	0.662
	Q37	0.760		Q45	0.499
	Q38	0.563	13.Evaluation	Q16	0.739
	Q39	0.755		Q17	0.526
	Q40	0.441		Q18	0.696
7. Regulation	Q09	0.518		Q19	0.662
	Q10	0.710		Q20	0.499
	Q11	0.557	14. Autonomy	Q29	0.438
	Q12	0.620		Q30	0.486
	Q13	0.736		Q31	0.465
	Q14	0.670		Q32	0.463
	Q15	0.567		Q33	0.608

### Confirmatory factor analysis

**Fig. 3:**
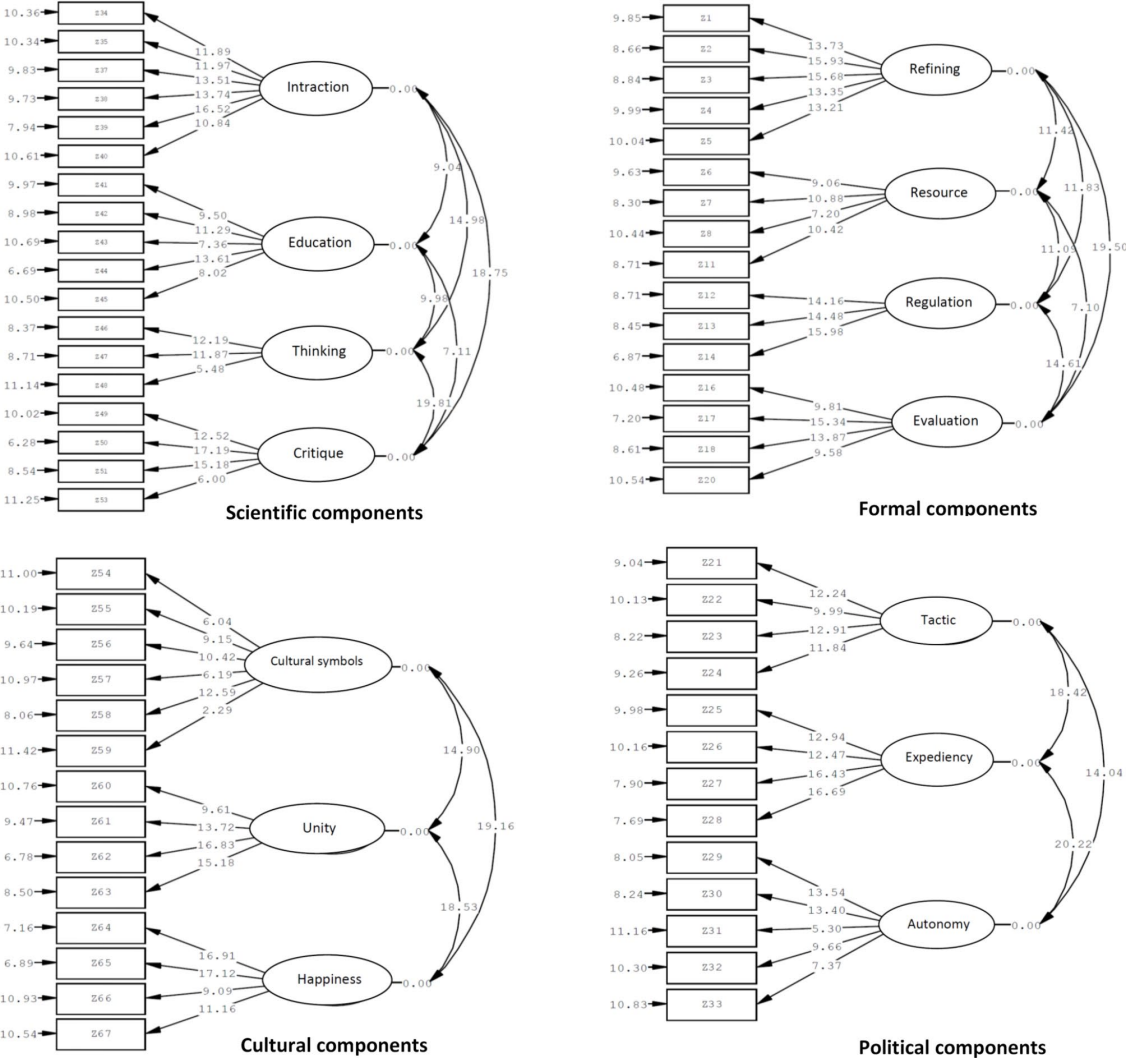
T-value of significant factor loading results for items of formal, political, cultural and scientific infrastructure of RASD questionnaire.

In the next step, using LISREL v8.80 software, confirmatory factor analysis was performed. In the confirmatory factor analysis, components were classified into four formal, cultural, scientific, and political categories. Based on the software’s recommendation, items 9, 10, 15, 19, 20, and 52 were removed. item 11 was transferred to the domain of resources based on the software’s suggestion. The fit indices for the second-order confirmatory factor analysis of the scientific development infrastructure were presented in Fig. 3.

Based on Fig. 3 and the results of the confirmatory factor analysis, the model’s fit has been confirmed at a statistically significant level.

Also, based on the CFA test indices, the results showed that the questionnaire had a good fit. The RMSEA, CFI, and GFI indices are expected to be equal to or greater than 0.90, which is desirable for all official, cultural, political, and scientific components, and all three indices are at the desired level. Also, the index K2 ratio to degree of freedom) is expected to be less than three, which is suitable for official (2.85), political (1.62), and scientific (2.16) indices, and is very close to 3 for the cultural index (3.11). In addition, the significant value in all indices is less than 0.001 (Table 9).

**Table 9:** Fit index for confirmatory factor analysis of RASD questionnaire.

Index	Observed values				Expected index^39,40^
	Formal	Political	Scientific	Cultural	
Chi-square	279.39	100.30	278.66	264.40	
Degrees of freedom (df)	98	62	129	84	
Chi-square to df ratio (χ²/ df)	2.85	1.62	2.16	3.11	≤ 3.00
Significance	*P* < 0.001	*P* < 0.001	*P* < 0.001	*P* < 0.001	≤ 0.05
Root mean square error of approximation (RMSEA)	0.08	0.04	0.06	0.09	≤ 0.10
Normal fit index (NFI)	0.94	0.97	0.94	0.93	≥ 0.90
Comparative fit index (CFI)	0.96	0.97	0.97	0.94	≥ 0.90
Goodness-of-fit index (GFI)	0.90	0.90	0.90	0.90	≥ 0.90

## Discussion

The results of this study can be categorized and interpreted in two parts. The first part focuses on identifying the components of infrastructure development and determining their contribution, while the second part examines the validity of the instrument and the importance of the components through factor analysis and psychometric evaluation.

To identify the components of RASD, the researchers initially extracted the components using semi-structured interviews and content analysis of expert opinions in higher education. Based on inductive content analysis and reflection, 67 codes (Items), 14 components, and 4 themes (concepts) were extracted. These components align somewhat with Bolman and Deal’s categorization of organizational development components into four general categories: structure, policy, human resources, and symbols^13,21,22^. In this study, 14 intermediary components were placed in four conceptual categories: formal, cultural, scientific, and political. In terms of the nature of the components, political and formal align with the structure and policy components, and the symbolic category of Bolman and Deal^13^ aligns perfectly with the cultural category in this study. In both categories, indicators such as rituals and ceremonies, festivals, the presence of pioneers, and factors that lead to unity and harmony in organizational perspectives are included^13,23^. Additionally, the scientific category in this study includes three components: critical space, intellectual and creative space development, scientific interactions, and education and empowerment of faculty members. This category falls under the human resource dimension in Bolman and Deal’s model^13,21^. In Bolman and Deal’s definition of human resources, motivation and abilities (knowledge, skills, and attitudes of individuals) are emphasized, which includes their scientific interest and ability^13,21,22,24^. In a similar model, Bush introduces a six-dimensional model^26^. Bush believes that the perspectives of educational leadership define their approaches and determine their instruments and methods. In Bush’s model, the politic culture, and formality components are similar to the components in our study, and the *Collegial dimension* is equivalent to the scientific dimension in our study, as it is based on expertise and knowledge and strengthened by scientific and specialized individuals^26^. Lorenz also considers nine essential elements for organizational development based on Bolman and Deal’s perspective: ecology, structure, politics, culture, psychology, human resource, functional, intelligence, decision making. Among these elements, four common elements are human resources, political, structural, and cultural^25^.

### Psychometric of RASD questionnaire

Face and content validity were used to validate the instrument. Additionally, reliability was confirmed using internal consistency and Cronbach’s alpha coefficient, and finally, construct validity was determined to explain the *" Requirements of Academic Space Development "* (RASD) infrastructure.

The results of the content validity analysis using CVI and CVR showed that the content validity was validated with an appropriate value. According to the Lawshe model, it is expected that with the survey of 10 experts, at least 62% agreement will be reached^32^. The average CVR value for the questionnaire was obtained to be very suitable, with most of the items having a score between 0.6 and 1, which is acceptable. In addition, the validity was also confirmed through CVI, which was proposed by Waltz & Bausell. To calculate the CVI, experts were asked to rate each item in terms of relevance, clarity, and simplicity on a scale of 1 to 4. Individuals who selected options 3 and 4 are divided by the total number of experts. If the resulting value is greater than 0.79, it is acceptable, but if it is between 0.70 and 0.79, the item needs to be reconsidered and revised^31^. The overall average CVI value for the questionnaire was obtained to be very high and reliable, with most of the items having a value of over 80%, and in some cases around 0.70.

The reliability of RASD questionnaire confirmed with internal consistency. Cronbach’s alpha coefficient is one of the most common tests for measuring internal consistency and determining reliability, especially in Likert scale questionnaires. The value of Cronbach’s alpha coefficient is between 0 and 1, and the closer the value is to 1, the greater the internal consistency with the dimensions of the questionnaire^35,36^. The greater the correlation between the questions, the higher the value of Cronbach’s alpha coefficient^37^. The overall reliability of the questionnaire is expected to be at least 0.70, and a value between 0.80 and 0.90 is excellent^36^. Generally, the overall reliability value exceeding 0.80 is excellent, and the closer this value gets to 1, the better the tool’s reliability^35,36,38^. Additionally, the researchers calculated the reliability by removing each of the questions to examine their correlation with the overall reliability (If item deleted). It is expected that by removing each question and calculating the reliability minus that question, the overall reliability value does not increase. This method is commonly used in reliability analysis to determine the consistency of the scale items with total reliability. Table 4 demonstrates that the correlation of each question with the overall reliability is desirable. Furthermore, the reliability of each component is tangible. The highest reliability value of the components is Refining (0.879), Expediency (0.864), Interaction (0.826), and Happiness (0.823), respectively. In most components, except for two cases, the reliability of the components is higher than 0.70, which is a suitable number, and two components have a reliability of about 0.60, which is relatively acceptable. It should be noted that the reason for the decrease in R of sub-components is that the reliability value is influenced by the number of questions, and therefore, the obtained values are suitable.

For construct validity, the *Total Variance Explained* showed four extracted factors that explain a total of 70.229% of the variance for Requirements of Academic Space Development. The amount of this variance and its consistency with the theory indicate the appropriate validity of the obtained measures. The KMO value and the significance of the Bartlett test show the adequacy of the sample. An acceptable KMO value for sample adequacy is approximately 0.70 to 0.79, and anything above that is excellent. The obtained KMO value of 0.897 indicates an appropriate sample size. Additionally, according to the obtained Sig value (*P* < 0.001) in the Bartlett test, the goodness of fit test is confirmed^34,39–41^. According to Table 6, the level of contribution of each variable was found to be greater than 0.6. The minimum expected value for this index is 0.5, and in some studies, a value of 0.4 or 0.3 is also acceptable. Therefore, a value > 0.6 is considered suitable. Another factor that confirms the construct validity is the data fit indices in confirmatory factor analysis. Based on the Table 9, a significant level of < 0.001 was obtained for each of the formal, political, scientific, and cultural domains. Additionally, the ratio of chi-square to degrees of freedom is expected to be less than or equal to 3. Based on the data fit indices in the formal, political, and scientific domains, this ratio was found to be suitable, while for the cultural domain, this ratio was reported as 3.11, which is somewhat close to three and relatively acceptable.

Furthermore, the RMSEA index is expected to be less than 0.1. It is suggested that values less than 0.05 are good, values between 0.05 and 0.08 are acceptable, values between 0.08 and 0.1 are conditionally acceptable, and values greater than 0.1 are unstable^42^. In the present study, (Table 9) the RMSEA scores obtained political component Is good (0.04), for scientific (0.06) and formal (0.08) and for cultural component (0.09) is conditionally acceptable^42^.

In addition, for the GFI index, a value greater than 0.9 is expected^43,44^. Based on the findings, this value was found to be desirable and acceptable for all components^40,42^. For the CFI index, a value greater than 0.9 is desirable, and in some cases, a value greater than 0.7 is also acceptable^45^. The results of this study indicate a desirable value for this index. Another index that confirms the model fit is NFI, which is expected to be ≥ 0.9 and was confirmed for all components^46^.

### Determination of component importance

As mentioned, the set of 14 factors extracted from factor analysis explains 70.229% of the RASD construct, and the top 5 factors cover about 50% of this value.

Based on the results of factor analysis, the underlying infrastructure that showed the highest factor load was scientific enthusiasm “*Happiness*”, with statements such as (positive reinforcement the hope and happiness, for the country’s progress, appreciation of esteemed elder professors and the existence of exemplary individuals in the university, etc.) showing the highest factor load compared to other areas. Scientific enthusiasm and pleasant feelings in the work environment are very effective factors in the success of faculty members and have significant effects on their performance and internal motivation^47^. The findings of Al-Bataineh et al.‘s research in 2021 also showed that the scientific enthusiasm of university professors has an important impact on improving the quality of university performance^48^. Scientific enthusiasm in universities is influenced by many factors^49^. In some studies, a significant relationship has been observed between the stresses of faculty members and the level of scientific enthusiasm^50^. The research by Zheng (2022) also showed that happiness, enthusiasm and positive feelings among faculty members are influenced by a collection of individual, psychological, occupational, and social factors^51^. Bolman and Deal^22^ describe cultural symbols as one of the most important infrastructures for development in universities. Schein also believes that organizing festivals, appreciating and giving importance to faculty members, and having pioneering individuals in the organization are cultural elements that have an impact on organizational development^23^. Part of the scientific enthusiasm of university professors comes from the feeling of importance and professional identity and the respect and status associated with their profession^52^. In addition, one of the most effective factors influencing scientific enthusiasm in universities is the sense of justice and fair treatment with regards to university privileges^53^. Research has shown that this issue has a profound impact on teachers’ sense of belonging to the organization and their organizational identification^54^.

As the results of the present study showed, the second component with the highest factor loading was “*Unity*”, which means the sense of academic identity, connection with other members and groups of the university, and importance to the overall excellence of the university. Essentially, unity refers to an individual’s sense of belonging to a larger group and to the surrounding community^55,56^. This concept refers to the extent to which a faculty member has organizational attachment and commitment and places their behaviors and roles not only for individual promotion but also for the development of the university and beyond that the community. This factor itself affects scientific enthusiasm, as research has shown that organizational enthusiasm and happiness, especially among knowledge workers such as university professors, is influenced by a sense of justice^54,57^, and this can be influential in the sense of belonging and solidarity with the university. It is noteworthy that the first and second components, which had the highest factor loading, were sub-factors of culture. The essence of culture is thought and knowledge and, according to Parsons, explains a set of ideas and values^58^ and has a close relationship with the element of “identity” and, like a solid framework, distinguishes human societies from each other and gives them identity and leads to the creation of belonging and convergence among them^59^.

The third factor extracted from the exploratory factor analysis in this study is “*Thinking”* The presence of an appropriate intellectual space and intellectual engagement in universities is the third priority identified by faculty members for the development of the academic environment. The items of this factor emphasize that universities should foster a culture of intellectual freedom, avoiding routine and habits, and promote free intellectual discourse within the university. This is particularly important because 21st-century universities are constantly faced with rapid and unpredictable changes, and faculty members and university administrators need to strengthen their thinking and reflection abilities to confront new events^60^. Multiple studies have highlighted the importance of strengthening critical thinking in universities and developing thinking skills among faculty members and administrators^60–62^. Creating a space for intellectual discourse and discussion can also be a catalyst for intellectual development.

The fourth factor is *“Refining”* which refers to improving quality mechanisms and feedback from the community. A review of the Table 3 shows that the items such as systematic communication of faculty activities with quality optimization programs, coordination of academic functions within the university to enhance quality, review and refinement of current laws and structures, and critique and review of academic functions through interaction with social institutions constitute the factor of Refining. Although this factor falls under the formal domain, it emphasizes the need for reviewing the academic process and mechanisms based on interaction and feedback from the surrounding environment. Goodyear (2022) defines a good university as one that prioritizes the quality development of universities based on feedback from the community and the relevance of curriculum, teaching, research activities, and other university functions based on community engagement^63^. Connell (2019) also identifies engagement as an important and necessary characteristic of a good university. In other words, one of the main tasks of universities is to observe the community and its new needs, which provides foresight and the ability for universities to face new changes^64^.

The fifth priority is the “*Expediency”* factor, which is a subset of political factors. This factor emphasizes the ability of leaders and managers to negotiate and communicate with the organization and present a positive image of the organization. Bolman and Deal (1997) describe political power as one of the essential elements of organizational development^22^. Tony Bush also defines a political approach as one of the organizational realities^26^. Masoudi et al. (2023) found in a study on the components of virtual education development that political factors such as the ability to negotiate and compromise in meetings and the presence of powerful individuals in organizations can be effective in organizational development^65^.

The first five factors collectively explain about 50% of the model fit. The next factors in descending order are: Interaction with items (34–40), Regulation (15 − 9), Cultural Symbols (59 − 54), Critiques (52 − 49), Tactics (25 − 21), Resources (8 − 6), Education (45 − 41), Evaluation (20 − 16) and Autonomy (33 − 29). Based on the extracted pattern from the confirmatory factor analysis, 14 components were identified based on the Bolman and Deal model in four themes or cultural, formal, scientific, and political concepts. This finding is highly compatible with the Bolman and Deal model^13,21,22^. The cultural and political patterns align completely with the Bolman and Deal model. The formal infrastructure in the current study is consistent and adaptable with the formal components in Bolman and Deal’s model. The human resource component is mentioned in the Bolman and Deal model, where the organization is seen as a “family.” Managers emphasize the importance of knowledge, skills, attitudes, emotions, and relationships of individuals, believing that the organization should meet its needs through facilitating support and empowerment of its members^24,65^. In the current study, the scientific components are referred to as education, interaction, thinking, and critique, with the common element in all these cases being human resources. Additionally, the cultural, political, and structural components in the Tony Bush model align perfectly with the present model, and the collaborative model also corresponds to the scientific approach in the current study^26^.

In a similar study on the factors influencing the development of MOOCs, Barzekar et al. (2023) identified four factors: political, cultural, human resources, and structural^66^. In another study comparing the current and desired states of these components, the results showed that the current state is significantly lower than the desired level in the cultural, structural, political, and human resource components. In examining the current state of the indicators, participants emphasized the necessity of cultural development, the organization of national festivals, encouragement of outstanding activities, fostering a sense of belonging and membership among faculty, and the existence of motivational mechanisms for faculty and staff^67^.

## Conclusion

Universities are complex environments, and the diversity of their functions in producing, transferring, and applying knowledge and providing specialized services increases their roles in responding to society’s new needs every day. The variable surrounding environment with rapid changes always challenges universities with new challenges. But the main pillar of university dynamism is maintaining and developing the scientific environment in universities and providing the necessary conditions for scientific development. This development is influenced by a set of organizational, cultural, human resources, and political factors. Universities need to develop cultural symbols. Culture, as a foundational factor, has an impact on the balanced and purposeful growth of other infrastructures. Cultural symbols are value-creating, create unity, and create vibrancy and are therefore at the forefront of development. On the other hand, the most important product produced in universities is science, this ancient heritage of scientific spaces. In this research the most important factor in the development of the academic environment is hope and happiness. This issue is strongly related to the faculty members’ motivation and how they perceive its importance in the academic environment. Among all the financial problems and facilities challenges and etc., what matters the most is the individual’s sense of self and happiness in the university environment. This is something that university officials should prioritize, especially considering that faculty members often neglect their own internal needs. Faculty members may not explicitly demand happiness from university managers, but their sense of dissatisfaction can have a slow and silent impact on the development of the academic environment. Therefore, it is important for university officials to prioritize the well-being of faculty members and provide them with a positive work environment. This includes promoting self-acceptance, positive relationships with others, and autonomy. Additionally, research has shown that an individual’s sense of unity with the university community is one of the most important factors in the development of the academic environment. Furthermore, an individual’s satisfaction with the academic environment, happiness, and hope directly affect their commitment to the organization. Therefore, universities should prioritize the well-being of their faculty members and provide them with a supportive and positive work environment to promote their happiness and satisfaction. In a nutshell, it can be said that our universities need a rethinking, a concept that has been addressed in this research as “re-architecture.” Perhaps the choice of the term “architecture” is appropriate because we also need structural changes in universities, while also paying attention to cultural factors. By combining science, re-engineering, cultural considerations, and an artistic integration of all factors influencing university development, we can achieve an appropriate model of scientific development space in universities. Also, in psychometric of research instrument, it seems the instrument developed to evaluate the Requirements of Academic Space Development has appropriate validity and reliability indices and could be used for future studies in higher education.

### Strength and limitations

#### Strength

This text discusses an assessment instrument for measuring the development of scientific space in universities. The instrument has been validated through various methods and has shown to be valid and reliable. The mixed and triangulated research method increases the credibility of the work. The research is innovative and exploratory in its approach to the topic of scientific space in universities.

#### Limitations

The research is new and has only been conducted in one university, so further testing in different environments is needed. Although the KMO test confirmed the adequacy of the sample, some sources recommend 10 times the number of variables due to the number of questionnaire items. Therefore, it is necessary to retest this questionnaire in larger populations.

## Data Availability

The datasets used and/or analyzed during the current study are available from the corresponding author on reasonable request.

## Abbreviations

RASD: Requirements of academic space development
EFA: Exploratory factor analysis
CFA: Confirmatory factor analysis
CVR: Content validity ratio
CVI: Content validity index
GFI: Goodness of fit index
NFI: Normed fit index
CFI: Comparative fit index
RMSEA: Root mean square error of approximation
